# The perspective of undergraduate dental students on web-based learning in pediatric dentistry during the COVID-19 pandemic: a Korean multicenter cross-sectional survey

**DOI:** 10.1186/s12909-021-02928-w

**Published:** 2021-09-25

**Authors:** Lan Herr, Myeong Kwan Jih, Jonghyun Shin, Yong Kwon Chae, Hyo-Seol Lee, Sung Chul Choi, Ok Hyung Nam

**Affiliations:** 1 Department of Dentistry, Graduate School, Kyung Hee University, Seoul, South Korea; 2 Department of Pediatric Dentistry, School of Dentistry, Chosun University, Gwangju, South Korea; 3 Department of Pediatric Dentistry, School of Dentistry, Dental and Life Science Institute, Pusan National University, Yangsan, South Korea; 4 Department of Pediatric Dentistry, School of Dentistry, Kyung Hee University, Kyungheedae-Ro 26, Dongdaemoon-Gu, Seoul, 02447 South Korea

**Keywords:** COVID-19, Dental education, Distance learning, Online learning, Pediatric dentistry

## Abstract

**Background:**

The COVID-19 pandemic changed the world and created a shift in the dental education program. This sudden change in the dental education program may have affected the academic standards of dental students. This study aimed to evaluate the overall satisfaction and effectiveness of online learning in pediatric dentistry of undergraduate dental students’ during the COVID-19 pandemic in South Korea.

**Methods:**

An anonymous online survey was sent to three dental schools, and responses were collected from dental school students. Questions included the demographics, perspectives of online classes, comparison of online and offline pediatric dentistry classes and opinions on how dental schools are handling the pandemic. Students’ perspectives on online classes were evaluated based on satisfaction with online education. Data were analyzed using the Kruskal-Wallis test and the Mann-Whitney U test.

**Results:**

Most students took online classes from home (80.9%) using Zoom (50.4%). The majority reported overall program satisfaction (74.1%) and agreed that universities implemented online classes well (55%). Students who were in favor of online classes responded more positively to questions on the effectiveness and safety of online learning (*p* < 0.05). Regardless of satisfaction with online education, the students agreed that the online education shift was the right decision in pandemic outbreak.

**Conclusions:**

Dental students in South Korea preferred and adapted well to the web-based learning program in pediatric dentistry during COVID-19 pandemic.

## Background

The COVID-19 pandemic has changed the world since its first outbreak on 31 December 2019 and impacted people from all walks of life. According to the World Health Organization (WHO), COVID-19 is a new respiratory disease caused by the coronavirus SARS-CoV-2 transmitted primarily by droplets of saliva, coughs, sneezes or exhales from an infected person [1]. According to WHO, as of August 2021, there are globally almost 200 million confirmed cases and over 4 million deaths. The acceleration of the number of confirmed cases and deaths as this global health threat continued to spread over, the need for development of safe and effective vaccines was urgent. To end COVID-19 pandemic, worldwide vaccination has been deployed in hopes of reaching herd immunity and as of August 2021, there were almost 4 billion vaccine doses administered [2]. Delta variant infections threaten herd immunity and being fully vaccinated does not necessarily perfectly immune one from the possibility of breakthrough infection [3] and therefore, one is still encouraged to take precautions and continue to wear masks, clean hands, and keep appropriate social distancing.

COVID-19 has caused the largest disruption of education systems in history. Closures of educational institutions have affected vulnerable children, youth, and people with disabilities; they have also impacted the lives of adults, employees, employers, and communities. Substantial education has moved to online learning through recorded or live lectures using online platforms [4].

Previous researches have been published in various journals about adapting to distance learning in medical and dental schools in response to the COVID-19 pandemic [5–9]. The corona crisis has also created a global paradigm shift in dental education [10–14]. Dental schools in Italy closed down due to COVID-19, with classes and examinations moving online [10]. The beginning of the semester had to be delayed to allow the teachers to prepare class recordings according to curriculum changes. Clinical training was replaced by online case presentations. Student clinic appointments were rescheduled and remained available only for the treatment of dental emergencies. A survey conducted in 21 dental education institutions in China, 33 online courses were offered 10 weeks before the epidemic began [15]. The number steeply rose to 119 online courses within 2 weeks of the epidemic. Research on education shifts in the field of dental medicine often focuses on student feedback on online classes [11, 15–18]. However, studies focusing on methods and access to online education or student perspectives on how well the school has handled the sudden education paradigm shift are insufficient.

Digital transformation is gaining immense attention in the medical field and implementing distance learning may bear a positive effect on future dental education even beyond COVID-19. However, the field of dental medicine mandates hand skills and clinical training. It is one area that cannot be transmitted by theoretical learning alone. There are concerns that clinical education cannot be substituted with distance learning, nor does online education verify student clinical skills [19]. Dental students need practical training in preclinical curriculum and patient treatment in the clinical curriculum. It is indicated that personal instruction and feedback cannot be replaced by online learning [20]. Pediatric dentistry is especially a dynamic field mandating person-to-person interactions which requires patient management practice [21]. Online education simply cannot convey the emotional support or mutual interaction which are especially key to successful treatment in treating pediatric patients. However, distance learning seems the only feasible option in this current pandemic era until population immunity is reached.

In the South Korean 4-year dental school education system, students take the theoretical courses for 4 years and start the preclinical curriculum and practical training in the third year and continue until the final fourth year [22]. In the latter half of 2019, the year before the pandemic outbreak, second year dental students attended pediatric dentistry class in classrooms, just like what normal classes would conventionally be. In the early half of 2020, the year COVID-19 hit, the same students now in third year took the next level pediatric dentistry class fully online. This class of students were chosen in this study as they allowed direct comparison of offline and online pediatric dentistry class as they experienced both methods in the same subject.

The purpose of this study was to compare the online and offline learning methods of pediatric dentistry of undergraduate dental school students in South Korea to evaluate the overall satisfaction and effectiveness of the new learning method. This paper also aimed to investigate the role of dental school in assimilating online education in response to COVID-19 pandemic.

## Methods

### Study participants

This study included undergraduate dental students from three dental schools in South Korea. Inclusion criteria for this study demanded the online curriculum design and full conversion to online education after COVID-19 outbreak and for these reasons, three dental schools were chosen. Third-year dental students in the year 2020 were specifically selected for this study as they completed an online pediatric dentistry class and conventional classroom learning in 2019 as second year students before COVID-19, which allowed a direct comparison between offline and online classes as they had experienced both in the same subject. Sample size was calculated using G*Power 3.1.9.7 (Heinrich Heine University Düsseldorf, Düsseldorf, Germany) and the number of study participants needed for this study was estimated at 0.90 power with a 5% margin of error [23]. The sample size needed was 207 students. This study proposal was reviewed and approved by the Ethics Committee of Kyung Hee University Dental Hospital, Kyung Hee University (KH-D20–034), Pusan National University Dental Hospital (PNUDH-2021-001), and Chosun University (2–1,041,055-AB-N-01-2020-69).

### Online survey

An anonymous online questionnaire was used via Google forms. The survey was distributed online via e-mail to 227 third-year dental students of three participating dental schools, and responses were collected for a week. Informed consent was obtained from every participant. There were 220 responses, yielding a 97% response rate. The questionnaire was developed to assess the demographic characteristics of study participants and student perspective of the online learning method using a 5-point Likert scale with modifications from a previously reported study [24]. The questions included a direct comparison of offline and online pediatric dentistry classes and the handling of COVID-19 pandemic and online learning by dental schools. The participants had to reflect back on the past experiences with offline learning. There were 36 questions in total; 7 demographic questions, 16 on satisfaction of different aspects of online learning, 9 on direct comparison of online and offline pediatric dentistry class and 4 on dental schools. The survey questions on online learning and dental schools were based on a 5-point Likert scale (Table 1).

**Table 1:** Online survey questionnaire

**Demographics**				
1. What is your gender?				
2. Who do you currently live with?				
3. Do you have any previous experience with online class? (does not have to be school related)				
4. Which platform was used for your online pediatric class?				
5. Was your online class live or pre-recorded?				
6. Which device did you use to take online class?				
7. Where did you take online class?				
**Online Classes**				
Rate your level of agreement with each statement according to Likert’s 5-point scale.				
Strongly Disagree	Disagree	Neutral	Agree	Strongly agree
1. Online classes were prepared and structured well.				
2. Online assessments and evaluations were efficient.				
3. Family’s reaction online classes was positive.				
4. I was overall satisfied with online classes.				
5. In current pandemic, a shift to online classes was a good option.				
6. I prefer online classes for the next semester.				
7. If safety guideline is assured, I would prefer conventional classes.				
8. Communication with professors was more convenient and effective in online classes.				
9. Communication with peers was more convenient and effective in online classes.				
10. Online classes helped me prepare better before the class.				
11. Online classes helped me review better after the class.				
12. Online classes stimulate and motivate self-directed learning.				
13. Online classes will create difficulties in the clinical aspects of dental learning in the future.				
14. Online classes will pose challenges in finishing dental school curriculum in the future.				
15. Online classes will create difficulties in preparing for the national board exam in the future.				
16. I am afraid that I will be a worse dentist than others who took conventional classes and clinical training.				
**Comparison of Online and Offline Pediatric Dentistry Class**				
Choose one of the following answers.				
Offline	Equivalent	Online		
1. Time efficiency				
2. Class participation and concentration				
3. Attendance rate				
4. Efficient knowledge transfer				
5. Efficient communication				
6. Efficient Question & Answer with professor				
7. Academic fun and enthusiasm				
8. Academic motivation				
9. Academic stress				
**Dental School/University**				
Rate your level of agreement with each statement according to Likert’s 5-point scale.				
Strongly Disagree	Disagree	Neutral	Agree	Strongly agree
1. The university is handling the current pandemic situation well.				
2. The university implemented online classes well.				
3. The university provided notifications and guideline beforehand to allow students prepare for online classes.				
4. The university is actively communicating with students and feedback is quick and constructive.				

### Group categorization

Study participants were asked overall satisfaction of online classes using a 5-point Likert scale. The authors decided to divide responses into three groups based on 5-point Likert scale. Students who strongly agreed or agreed on overall satisfaction of online classes were considered positive towards online classes. Group 1 consisted of students who showed positive responses to online classes and selected “Strongly Agreed” or “Agreed”; Group 2 consisted of “Neutral” students; Group 3 consisted of students who showed negative response to online classes and selected “Strongly Disagreed” or “Disagreed”.

(i) Group 1: participants who were in favor of online classes (*n* = 163)
(ii) Group 2: participants who remained neutral (*n* = 50)
(iii) Group 3: participants who were not in favor of online classes (*n* = 7)

### Statistical analysis

Data were analyzed using the SPSS version 20.0 (SPSS Inc., Chicago, IL, USA). The Shapiro-Wilk test was performed to verify the normality of the data. After the normality test, the Kruskal-Wallis test was used to analyze data (*p* < 0.05); the Mann-Whitney U test was used for post-hoc analysis (Bonferroni correction, *p* < 0.0167).

## Results

### Characteristics of study participants

Characteristics of study participants are shown in Table 2. There were more male participants (60.9%) than females. Most students lived alone (73.6%). Zoom was the most used platform for online classes (50.4%), followed by the school’s website (32.1%). Pediatric dentistry online class was live (63.6%). Most students used laptops (35.5%) or tablet computers (27.5%) to participate in online classes. Students took their online classes mostly from home (80.9%).

**Table 2:** Characteristics of student participants

	Frequency	
	N	%
**Gender**		
Male	134	60.9
Female	86	39.1
**I currently live with**		
Alone	162	73.6
Family	38	17.3
A roommate	19	8.6
Roommates	1	0.5
**I have previous experience with online classes/webinar**		
Yes	210	95.5
No	10	4.5
**I use this platform for online classes**^a^		
Zoom	207	50.4
School’s own website	132	32.1
Google	71	17.3
YouTube	1	0.2
**Pediatric dentistry online classes were**		
Live	140	63.6
Pre-recorded	80	36.4
**I participated in online classes on**^a^		
Laptop	123	35.5
Tablet computers	95	27.5
Cell phones	73	21.1
Desktop computers	55	15.9
**The location where I took online classes was**^a^		
Home	212	80.9
School	20	7.6
Café	19	7.3
Public transportation (car, bus, subway, taxi)	11	4.2

^a^These questions were allowed to select more than one answer

### Overview of dental students’ perspectives on online classes

**Fig. 1:**
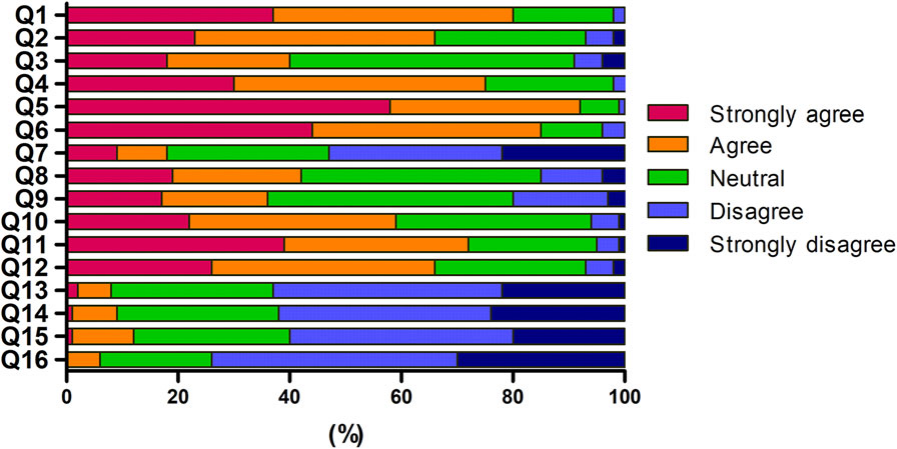
Students’ responses to questionnaires on opinions about online classes. Responses are based on a 5-point Likert scale. The corresponding questions are as follows: Q1. Online classes were prepared and structured well; Q2. Online assessments and evaluations were efficient; Q3. Family’s reaction to online classes was positive; Q4. I was overall satisfied with online classes; Q5. In the current pandemic, a shift to online classes was a good option; Q6. I prefer online classes for the next semester; Q7. If safety guideline is assured, I would prefer conventional classes; Q8. Communication with professors was more convenient and effective in online classes; Q9. Communication with peers was more convenient and effective in online classes; Q10. Online classes helped me prepare better before the class; Q11. Online classes helped me review better after the class; Q12. Online classes stimulate and motivate self-directed learning; Q13. Online classes will create difficulties in the clinical aspects of dental learning in the future; Q14. Online classes will pose challenges in finishing dental school curriculum in the future; Q15. Online classes will create difficulties in preparing for the national board exam in the future; Q16. I am afraid that I will be a worse dentist than others who took conventional classes and clinical training

Figure 1 shows an overview of the responses to online classes. Most students reported overall satisfaction (74.1%) with online classes. Given the current COVID-19 pandemic, 92.3% of students agreed that a shift to online classes was an appropriate decision, and 85% of students preferred to continue online learning for the next semester. The majority of students (61.9%) disagreed on facing difficulties graduating dental school if COVID-19 lasted longer than expected, and 60% disagreed on experiencing challenges preparing for the national board exam.

### Dental students’ perspectives based on satisfaction with online classes

**Fig. 2:**
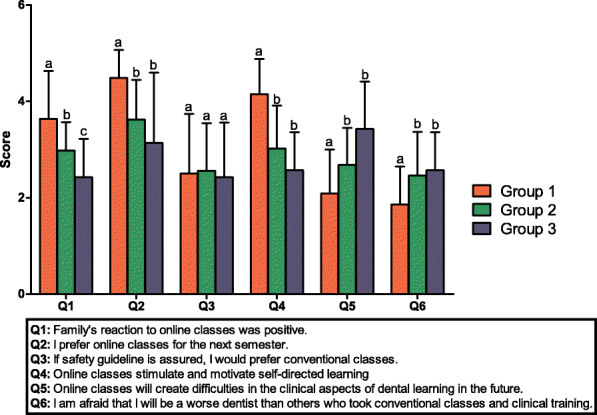
Students’ responses based on satisfaction with online classes. Group 1 consisted of students who answered “Mostly agree” and “Agree.” Group 2 consisted of students who answered “Neutral.” Group 3 consisted of students who answered “Disagree” and “Mostly disagree.” The scores were presented as a 5-point Likert scale. The group differences were evaluated by the Kruskal-Wallis test and Mann-Whitney U test, post-hoc analysis. Different case letters indicate statistically difference (*p* < 0.0167)

Group 1 participants offered more positive responses than Group 2 and 3 subjects, except on the question related to online learning preferences for the next semester (Fig. 2). Between Groups 2 and 3, Group 3 participants offered more negative responses to the question about the family’s positive reaction to online classes (*p* = 0.016).

### Comparison of online and offline pediatric dentistry class

**Fig. 3:**
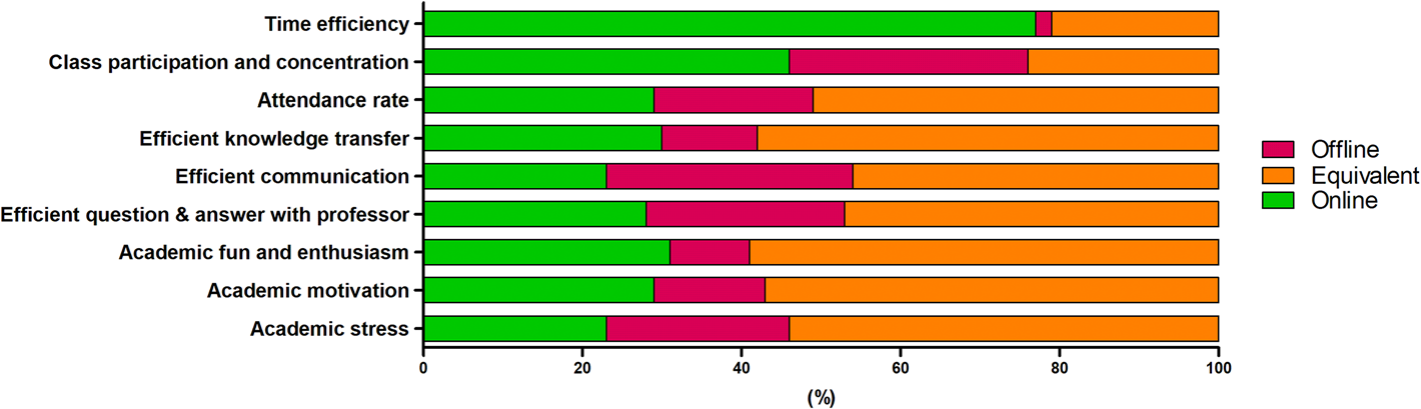
Direct comparison of offline and online pediatric dentistry class. Students replied online class was more time efficient (76.4%) and hold better class participation and concentration (46.4%)

Figure 3 shows a direct comparison of online and offline pediatric dentistry class. Most students felt offline and online classes to be equivalent in most criteria. Time efficiency (76.4%) and class participation and concentration (46.4%) were two parameters students’ preferred online classes for.

### Dental students’ perspectives on how the dental school was operating online classes

**Fig. 4:**
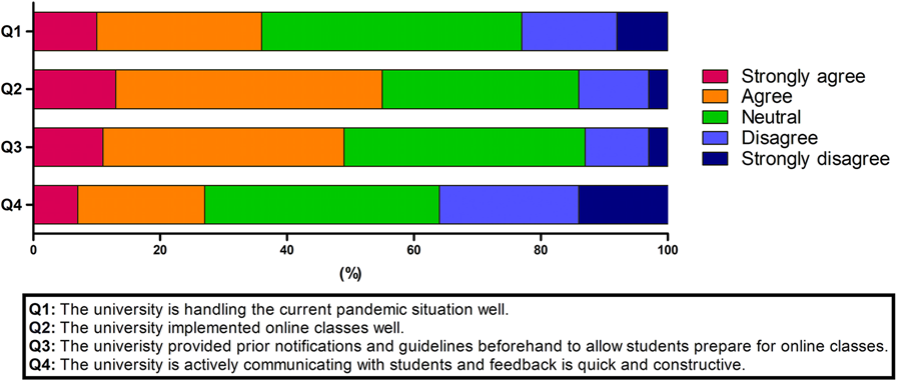
Students’ responses to questionnaires on how well the dental school is coping with the COVID-19 pandemic. On average, 41% of participants reported positive responses (“agree” or “strongly agree” based on a 5-point Likert scale) for all questions

The results are presented in Fig. 4. Most dental students (55%) agreed that the university implemented online classes well and provided prior notifications and guidelines to allow the students to prepare for online classes (49.1%). Students remained neutral on their opinions on active communication and constructive feedback between the university and students (36.8%). 35.9% of the students felt that their dental school was dealing well with the current pandemic situation.

## Discussion

The COVID-19 pandemic changed various aspects of people’s lives not only on the health and socio-economic conditions but also on the education spectrum. Dentistry is one field not immune to contagious respiratory diseases because of everyday uses of aerosols, rotary hand pieces, and contact with saliva and mucosa. To be part of social distancing in measures to protection from spread of the corona virus, dental education had a paradigm shift from conventional to online curriculum in the past year. Students, professors, training faculties, and dental schools were unfamiliar with this new educational realm that provided a short preparation time. It is valuable to exchange experiences in dental education from different countries to cope with this pandemic [25–28]. This study aimed to investigate undergraduate dental student perspectives on web-based learning in pediatric dentistry during the COVID-19 pandemic in South Korea and compare them to conventional learning methods using online questionnaire to evaluate the overall satisfaction and effectiveness of the new learning method.

In this study, the most used online platform was ZOOM, a finding in agreement with previous studies [29, 30]. The most used hardware devices for online classes were laptops, followed by tablet computers and cell phones. This finding was in agreement with a study conducted on German dental students, in which the majority of students used laptops (69.8%), tablets (16.5%), and smartphones (7%) [31]. This result was also confirmed by a previous study on Brazilian dental students [32]. This finding may be related to student familiarity with using devices for daily use. Most dental students today have internet access and basic skills to operate mobile devices [33]. The majority of students accessed online education from home, a finding consistent with another study showing that the highest participation was achieved in the bedroom, followed by the living room and the dining room [32]. This finding aptly represents the currently recommended self-quarantine period.

Most Korean students reported overall satisfaction with online classes. They also felt that the shift to online classes was a good option, considering the current pandemic. Homogenous studies conducted in different parts of the world such as Brazil [34], Chile [35], Italy [36], Iraq [37], Jordan [30], Germany [31], and China [15] showed similar results. Students worldwide well-adapted to the new online dental education delivery. Korean students perceived a lack of communication with professors in online classes, whereas they did not feel a lack of communication with classmates, probably due to easier approachability, less pressure to contact one’s peers than professors, and prompt feedback from fellow students. Moreover, Nelson et al. [21] suggested that the teacher’s virtual office hours be scheduled in the coming year to provide an opportunity for missed in-person interactions. In Korean dental education, students take the national board exam at the end of the fourth year. Students in this study will be taking the national board exam the very next year and without definitive end to the pandemic assured, online education endures significant effect in preparing for the national board exam. More than half of the students disagreed that online classes could create difficulties in finishing dental school in time or preparing for the national board exam in the future. This result was consistent with a previous study, suggesting that less than half of the students were concerned about completing the degree program on time and passing the national board exam [38]. A newly introduced online high-stake exam was successfully implemented to final year dental students [39] and this maybe the new method of assessment and examination should COVID-19 continue however, clinical evaluation remains to be solved.

Results suggest that the level of satisfaction is associated with the effectiveness and safety of online classes. Family’s reaction to online classes were significantly positive in group 1 compared to group 2 and 3. The students mostly worried about their family members as dental clinical training exhibits a higher risk of contracting COVID-19 infection than other courses in medical field. Studies showed that the participants were concerned about the well-being of the family [36] and troubled with the idea that they could be a possible route of transmitting the infection to older family members [38]. If the necessitates for personal protection and actionable guidelines for sanitation of the clinical environment not be provided, it would not be surprising if students, practitioners, trainers, and of course patients are reluctant to visit dental clinics [29].

Group 1 responded more positively to questions on the effectiveness of online classes than other groups. A similar study categorized participants according to satisfaction levels and showed that the satisfied group responded more positively to questions related to active instructor participation in discussions, multimedia use, and less time investment [30]. Alike findings indicate that students showed positive attitude towards online curriculum.

In direct comparison of online and offline pediatric dentistry classes, students strongly agreed online class was time efficient and better for class participation and concentration. Other parameters said otherwise. Equivalent was the most chosen answer for academic stress and motivation or means of effective communication or efficient knowledge transfer. When students are asked opinions about online classes based on pandemic outbreak, many answers are amicable toward online education. However, this feedback may rely heavily on the current widespread epidemic and maybe biased but not entirely on the online education itself. The environmental factor is inevitably playing a major role in the current wave of infectious disease. When the focus of questions is highlighted on education, answers vary. Students do not hang back from online education and welcome the idea; however, there exists realistic limits on conveyance of dental knowledge via web. In regards to dental education, online learning has its ups and downs. This is even more so applicable in pediatric dentistry. It is one field in dentistry mandating person-to-person interactions with patient management. Not only is the mutual interaction important between the doctor and the pediatric patient, but also between the doctor and the parents/guardians. This simply cannot be conveyed online or via web. This needs practical practicing in real-life. When students are asked opinions solely based on education, students do not disfavor online education, but its preference is not more notable.

Students and lecturers are important components of the online curriculum. However, a strong foundation is needed to stand by them, the dental school [40]. The dental students in this study thought that the universities handled the pandemic well and successfully implemented online classes. During the COVID-19 pandemic, students and faculty members were new to online education. Moreover, the shift was unexpected and required swift implementation. This sudden unforeseen situation caused anxiety and emotional stress in both parties [32, 35, 38]. Earlier during pandemic, students may have enjoyed the sudden free time because they could stay home instead of coming to school to attend class. Faculty may have appreciated the family time they were given however, the overwhelmed feeling with the new circumstances and academic load must have been a burden in both staff and students. In this manner, the institution should implement support system not only on academic delivery but also the emotional stress of community members. For coping with the pandemic, it is of utmost importance that dental students, as future clinicians, be equipped with reliable and accurate medical and pandemic information for patient management and cooperation. Furthermore, dental schools should provide in-depth explanations to avoid misunderstanding [34, 36].

This study is limited to only 3 Korean dental schools but responses may be different for students in schools more adversely affected by COVID-19 and with different online curriculum design. The current findings represent very short time frame as recommendation and guidelines are adjusted according to pandemic evolvement and vaccine development. However, this study highlighted perspective of Korean dental undergraduate students on online learning during COVID-19 pandemic. Further studies should elucidate on psychological reactions such as emotional stress and quality of life and institutional endeavor to shed light in students’ knowledge in epidemics.

## Conclusions

This study highlighted undergraduate dental student perspectives on online learning especially in pediatric dentistry during the COVID-19 pandemic in South Korea compared to conventional learning methods. Dental students in South Korea preferred and adapted well to the web-based learning program in pediatric dentistry.

## Data Availability

All data generated or analyzed from this study are included in this published article.

## Abbreviations

COVID-19: 2019 novel coronavirus
WHO: World Health Organization
